# Simultaneous Binding of the Anti-Cancer IgM Monoclonal Antibody PAT-SM6 to Low Density Lipoproteins and GRP78

**DOI:** 10.1371/journal.pone.0061239

**Published:** 2013-04-19

**Authors:** Zachary Rosenes, Yee-Foong Mok, Shuo Yang, Michael D. W. Griffin, Terrence D. Mulhern, Danny M. Hatters, Frank Hensel, Geoffrey J. Howlett

**Affiliations:** 1 Department of Biochemistry and Molecular Biology, Bio21 Molecular Science and Biotechnology Institute, University of Melbourne, Parkville, Victoria, Australia; 2 Patrys GmbH, Friedrich-Bergius-Ring 15, Würzburg, Germany; The Scripps Research Institute and Sorrento Therapeutics, Inc., United States of America

## Abstract

The tumour-derived monoclonal IgM antibody PAT-SM6 specifically kills malignant cells by an apoptotic mechanism linked to the excessive uptake of plasma lipids. The mechanism is postulated to occur via the multi-point attachment of PAT-SM6 to the unfolded protein response regulator GRP78, located on the surface of tumour cells, coupled to the simultaneous binding of plasma low density lipoprotein (LDL). We prepared and characterised LDL and oxidized LDL using sedimentation velocity and small-angle X-ray scattering (SAXS) analysis. Enzyme-linked immunosorbent (ELISA) techniques indicated apparent dissociation constants of approximately 20 nM for the binding of LDL or oxidized LDL to PAT-SM6. ELISA experiments showed cross competition with LDL inhibiting PAT-SM6 binding to immobilised GRP78, while, in the reverse experiment, GRP78 inhibited PAT-SM6 binding to immobilized LDL. In contrast to the results of the ELISA experiments, sedimentation velocity experiments indicated relatively weak interactions between LDL and PAT-SM6, suggesting immunoabsorbance to the microtiter plate is driven by an avidity-based binding mechanism. The importance of avidity and the multipoint attachment of antigens to PAT-SM6 was further investigated using antigen-coated polystyrene beads. Absorption of GRP78 or LDL to polystyrene microspheres led to an increase in the inhibition of PAT-SM6 binding to microtiter plates coated with GRP78 or LDL, respectively. These results support the hypothesis that the biological action of PAT-SM6 in tumour cell apoptosis depends on the multivalent nature of PAT-SM6 and the ability to interact simultaneously with LDL and multiple GRP78 molecules clustered on the tumour cell surface.

## Introduction

The human IgM monoclonal antibody, PAT-SM6, derived from human tumour tissue [1], is a potential anti-cancer agent capable of inducing tumour cell apoptosis in pre-clinical models of human cancer [2]. While the precise mechanism of PAT-SM6 induced cell death is not known, the process is accompanied by intracellular lipid accumulation leading to the hypothesis that PAT-SM6 facilitates the uptake of plasma lipids. Consistent with this proposal is the observation that both low density lipoproteins (LDL) and oxidized LDL interact with PAT-SM6 and enhance PAT-SM6-induced apoptosis [2]. PAT-SM6 also binds to the unfolded protein response regulator GRP78, which is over-expressed externally on the cell surface of tumour cells [3]. GRP78, also known as BiP (immunoglobulin heavy-chain binding protein), is a member of the heat-shock protein 70 (HSP70) family that prevents stress-induced apoptosis. Enzyme-linked immunosorbent assays (ELISAs), using different concentrations of coating GRP78, have previously established a high avidity interaction between PAT-SM6 and GRP78 mediated by the multi-point attachment of PAT-SM6 to GRP78 clustered on the surface of the microtiter tray [4]. In the present study, we investigated the avidity of the interactions of PAT-SM6 with LDL and oxidized LDL and the competitive nature of the binding of LDL and GRP78 to PAT-SM6. The role of multivalency in the interactions of PAT-SM6 with target antigens was investigated using antigen-coated polystyrene beads. The results demonstrate the importance of antigen clustering in generating the high avidities that characterize PAT-SM6 antigen interactions. The ability of PAT-SM6 to bind both GRP78 and LDL is consistent with the proposal that PAT-SM6 kills cells by delivering excess lipid in the form of LDL into tumours by binding simultaneously to GRP78 present on the surface of tumour cells [2].

## Experimental Procedures

### Materials

The human monoclonal antibody PAT-SM6 were expressed and purified from stable suspension cultures of a human cell line in serum-free media [5], [6]. Isotype control IgM was obtained from Jackson ImmunoResearch Labs, inc, West Grove, PA. PAT-SM6 was labelled with fluorescein using fluorescein isothiocyanate (Sigma-Aldrich) according to the manufacturer’s instructions. Mature human GRP78 containing a C-terminal 6× His-tag was expressed and purified from *E. coli* as described previously [4].

### Ethics Statement

Fresh human blood samples were obtained from St Vincent’s Hospital, Melbourne using protocols approved by the St Vincent’s Hospital Melbourne Human Research Ethics Committee. Written informed consent from participants was obtained for the original human work that produced the blood samples for the isolation of LDL. LDL was isolated by density fractionation preparative ultracentrifugation using KBr [7]. Oxidized LDL was prepared by the addition of 20 µM CuSO_4_ to 200 µg/mL LDL and incubation at room temperature for 16 hours [8]. Oxidation was monitored by measuring the absorbance at 234 nm and by thioflavin T (ThT) fluorescence. ThT was added to LDL to a final concentration of 8 µM and the fluorescence measured using a *f_max_* platereader (Molecular Devices, Sunnyvale, CA) equipped with 444/485 nm excitation/emission filters. Oxidation was stopped by the addition of EDTA to a final concentration of 1 mM. LDL and oxidized LDL were dialysed into phosphate buffered saline (PBS; 20 mM sodium phosphate, 150 mM NaCl, pH 7.4) containing 1 mM EDTA and 0.1% NaN_3_ and stored at 4°C. The protein concentration of LDL was determined from the absorbance at 280 nm corrected for light scattering [9] and using an extinction coefficient of 844 cm^2^/g estimated from the amino acid composition of apolipoprotein B. The molar concentration of LDL was calculated assuming a value of 2.2×10^6^ for the average molecular weight of human LDL [10].

### Small-Angle X-Ray Scattering (SAXS)

Synchrotron SAXS with inline size exclusion chromatography (SEC-SAXS) of LDL and oxidized LDL was performed at the Australian Synchrotron SAXS/WAXS beamline. Samples were subjected to SEC on a column with a pore size of 500 Å (WTC-050N5, Wyatt Technology, Santa Barbara, CA), giving an effective protein MW separation range of 15,000 to 5,000,000 Da. The column was equilibrated in buffer containing PBS at a flow rate of 0.4 mL/min. Injections were 20 µL with a protein concentration of 3 mg/ml. The beam size at the sample was 250 µm horizontal × 150 µm vertical (FWHM). Pilatus 1M detector images were analysed as averages of ten sequential 2 s exposures and converted to individual *I*(*q*) SAXS profiles using the Scatterbrain software (Australian Synchrotron). *I*(*q*) is the scattered X-ray intensity as a function of the magnitude of the momentum transfer vector *q* = (4πsinθ)/λ, where the scattering angle is 2θ and the X-ray wavelength is λ (1.12713 Å). Data were collected at 298 K using a camera length of 3.3 m, which allowed intensities to be collected over a *q-*range of 0.00429–0.24575 Å^−1^. SAXS profiles were analysed using the ATSAS (version 2.4) suite of programs [11].

### Sedimentation Velocity Analysis

Sedimentation velocity experiments using absorbance optics were conducted using an XL-I analytical ultracentrifuge (Beckman Coulter, Fullerton, CA) equipped with an An-60 Ti rotor at 20°C. Protein samples were added to double-sector epon-filled centrepieces with PBS in the reference compartment. Radial absorbance data were acquired at a rotor speed of 28,000 rpm, using a wavelength of 280 nm, and with radial increments of 0.003 cm in continuous scanning mode. Sedimentation velocity experiments were also performed using an XL-A analytical ultracentrifuge equipped with a fluorescence detection system (FDS; Aviv Biomedical) to monitor the sedimentation of fluorescein-labelled PAT-SM6 and KBPA-101. The sedimenting boundaries were fitted to a model assuming a distribution of sedimentation coefficients for non-interacting species, c(S), using the program SEDFIT [12]. Data were fitted using maximum entropy regularization, a P-value of 0.95 and a frictional ratio of 1.9 [4]. Sedimentation coefficients were corrected for the effects of LDL on solution density and viscosity, assuming values of 0.967 mL/g and 3.4 mL/g for the partial specific volume and intrinsic viscosity of LDL, respectively [10].

### Enzyme Linked Immunosorbant Assay (ELISA)

Antigens were coated onto 96-well microtiter trays (nunc maxisorp) by incubation overnight at 4°C. Plates were then washed 3 times in PBS, 3 times in PBS +0.1% Tween 20, 3 times in PBS and blocked with 5% (w/v) BSA in PBS for 1 hour at room temperature, then washed again as above. All antibody preparations were made in 5% (w/v) BSA in PBS. For indirect ELISA experiments, antibodies were applied directly to the plate and incubated for 1 hour. For competitive ELISAs, antibodies were incubated in solution with various amounts of antigen prior to application to the microtiter plate. After washing again as above, peroxidise-conjugated rabbit anti-human IgM secondary antibody (Dako) was applied to the plate and incubated for 1 hour according to the manufacturer’s instructions. After a final wash, antibody binding was determined using 100 µL per well of 3,3′,5,5′-tetramethylbenzidine substrate solution (Sigma) and measuring colour change at 655 nm. The time course for colour development was essentially linear over the optical density range 0–3. Measurements were taken 15 min after the addition of substrate. In control experiments, involving direct coating of the plates with PAT-SM6, over the concentration range 0–2.5 µg/mL, a systematic increase was observed in the ELISA signal with increased coating concentration, implying a direct correlation between the ELISA signal and the amount of antibody bound to the plate. ELISA data for the titration of primary antibody binding to immobilized LDL was analysed assuming a simple Langmuir binding isotherm described by the relationship Y = K_a_/(1−K_a_) where Y is the magnitude of the ELISA signal and K_a_ is the apparent binding constant, assuming a single class of binding sites.

### Antigen-coating of Polystyrene Microspheres

Suspensions of 1 µm diameter polystyrene microspheres (Polysciences, Inc) at a concentration of 2.5% (w/v) were washed by repeated pelleting (×3) in a microfuge and resuspending in PBS, PBS-0.1% Tween 20, and then a final time with PBS. The washed microspheres were then pelleted and resuspended in 2 mg/mL of antigen (LDL or GRP78) or 5% (w/v) BSA (blocking solution) to a final microsphere concentration of 2.5% (w/v). Tubes of microsphere/antigen mixture were incubated overnight on a rotating platform at 4°C. To determine the degree of antigen binding, the microspheres were pelleted, and the absorbance in the supernatant measured at 280 nm and compared to the absorbance of the original antigen solution. The coated microspheres were then washed as above and then blocked using 5% BSA and stored at 4°C until ready for use.

## Results

### Oxidation of LDL

**Figure 1:**
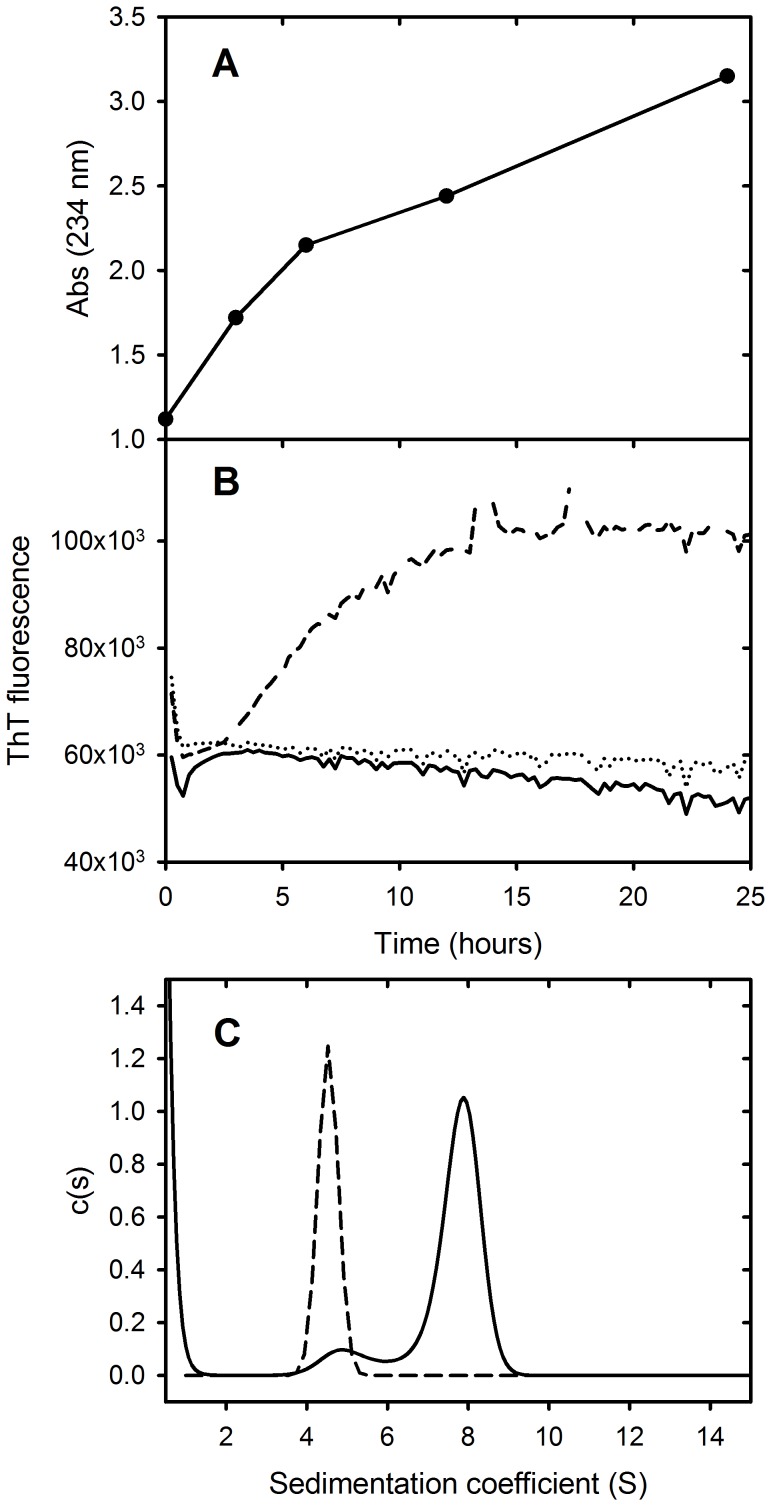
Cu^2+^-induced oxidation of LDL. (A) Change in absorbance at 234 nm as a function of incubation time. (B) Time course for the change in ThT fluorescence of LDL (solid line), LDL incubated with 20 mM CuSO_4_ (dashed line) or PBS alone (dotted line). (C) Sedimentation coefficient distribution obtained from sedimentation velocity analysis of LDL (dashed line) and oxidized LDL (solid line).

The Cu^2+^ induced oxidation of LDL was monitored by measuring the light absorbance at 234 nm (Figure 1A). The increase in absorbance at 234 nm approaches a maximum after 24 hours and is attributable to the formation of conjugated dienes [13]. LDL oxidation was also monitored by a continuous ThT assay [8]. The results show that ThT fluorescence of LDL incubated with 20 mM CuSO_4_ increases over the same time period reaching a plateau after 16 hours with no corresponding changes observed for control incubations of LDL or ThT alone. Further characterisation of oxidized LDL was carried out using sedimentation velocity analysis. Sedimentation coefficient distributions (Figure 1C) showed a single symmetrical peak for LDL with a modal sedimentation coefficient of approximately 4.5 S, while the distribution obtained for oxidized LDL was bi-modal with the major peak sedimenting with a modal sedimentation coefficient of approximately 8 S. This increase in sedimentation rate is attributable to a change in particle density due to a significant loss of lipid following oxidation [8].

### Small-angle X-ray Scattering (SAXS) Analysis of LDL and Oxidized LDL

SAXS analysis has been used extensively to characterise LDL and Cu^2+^-treated oxidized LDL [14], [15], [16]. This technique provides information on the overall size and shape of the lipoprotein particles from parameters such as the radius of gyration (*R*_g_) and the maximum dimension (*D*_max_), as well as information on the internal structure of the particles from the pair distance distribution function *P*(*r*). Here, LDL and oxidized LDL were subjected to synchrotron size-exclusion chromatography SAXS (SEC-SAXS) [4]. This method improves the quality of SAXS data by delivering the size fractionated the sample directly into the beam, and thus separating the scattering from the particle of interest from that of any aggregates, but also providing a near perfect buffer match for subtraction in the data reduction process.

**Figure 2:**
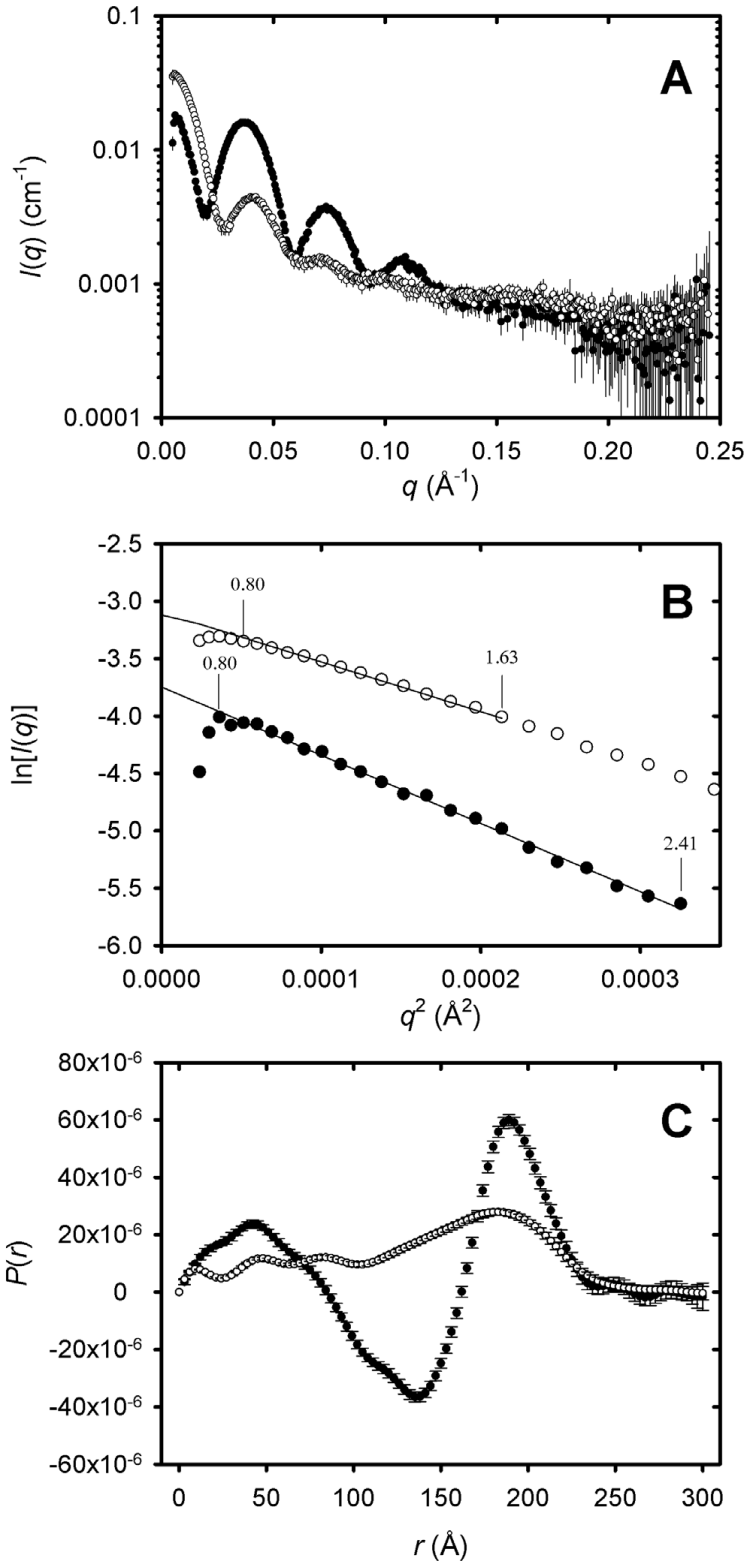
SAXS analysis of LDL and oxidized LDL. A. The SAXS data are shown as mean intensity *I*(*q*) ±1 standard deviation, as a function of momentum transfer *q* for LDL (solid circles) and oxidized LDL (open circles). (B) Guinier plots of the SAXS data at low *q* for LDL (solid circles) and oxidized LDL (open circles). The lower and upper *q*.*R*_g_ limits for the Guinier analyses are indicated. (C) Pair distance distribution function *P*(*r*) as a function of radial distance *r* for LDL (closed circles) and oxidized LDL (open circles).

The elution of LDL and oxidized LDL from the column was monitored by absorbance at 280 nm and SAXS intensity at zero-angle (*I*(0)) as a function of volume. The SAXS profiles of the peak species are shown in Figure 2A. For each species, *R*_g_ was calculated from the slope of their respective Guinier plots (ln[*I*(*q*)] vs. *q* ^2^) (Figure 2B). For LDL the Guinier plot was linear over a *q*.*R*_g_ range of 0.8–2.4, giving an *R*_g_ value of 134±1 Å. The *R*_g_ of the peak LDL species measured here is in good agreement with the published mean value of 139 Å from the range 121–160 Å observed for 17 LDL preparations from 12 donors [16]. For oxidized LDL the Guinier plot was linear over a *q*.*R*_g_ range of 0.8–1.6 giving an *R*_g_ value of 112±3 Å. Previous X-ray and neutron scattering studies of LDL oxidation employed static samples, which showed time-dependent and [Cu^2+^]-dependent increases in apparent *R*_g_, up to ∼160 Å, due the formation of aggregates [16], [17]. The SEC-SAXS derived *R*_g_ value reported here is the first measurement for isolated oxidized LDL particles in the absence of aggregates. For each SAXS profile the *P*(*r*) function was estimated by indirect Fourier transform (Figure 2C). The *P*(*r*) analysis indicated that LDL and oxidized LDL had similar *D*_max_ values of ∼250 Å, which is consistent with previous measurements [16]. The *R*_g_ value for LDL from *P*(*r*) analysis was 131±2 Å and for oxidized LDL was 109±3 Å, which are in good agreement with the values from the Guinier analyses. Cu^2+^-mediated oxidation causes characteristic changes in the internal organisation of the LDL particle, with has been attributed to a loss of ordered cholesterol ester and triacyglycerol structure [16]. *P*(*r*) analysis is diagnostic of this change, as the *P*(*r*) curve of LDL exhibits a strong negative peak at *r* = 135 Å, which is completely lost upon oxidation (Figure 2C). It has been proposed that this negative peak is due to negative contrast of the ordered hydrocarbon chains in the outer monolayer of LDL, which have lower electron density than the surrounding solvent. Oxidation is thought to result in the addition of oxygen to the unsaturated acyl chains, making them more electron dense than water and resulting in positive contrast [16], [17].

### Enzyme-linked Immunosorbent Assays of PAT-SM6 Interactions with LDL and Oxidized LDL

**Figure 3:**
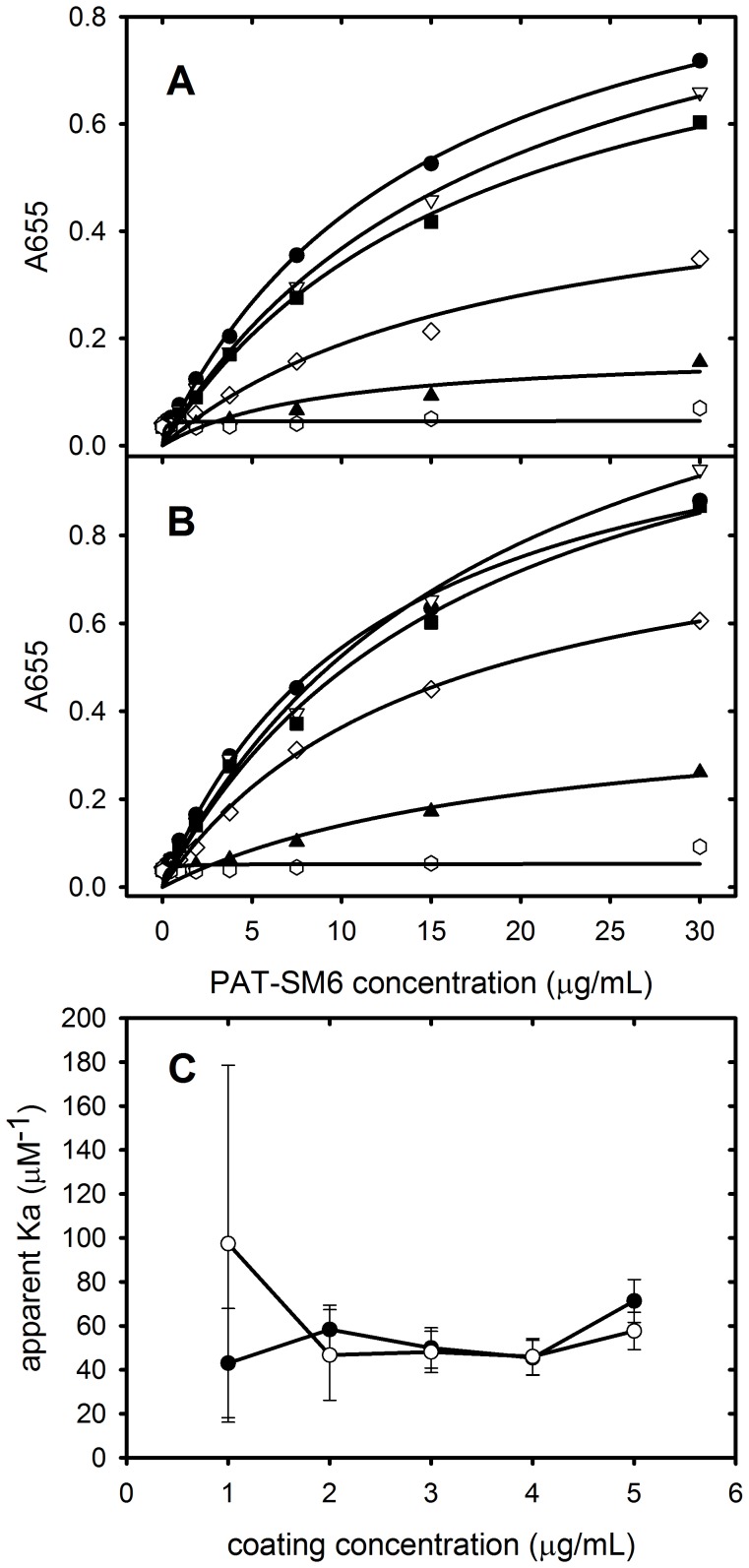
ELISA analysis of PAT-SM6 interactions. Assays were performed using (A) LDL or (B) oxidized LDL at coating concentrations of 0 µg/mL (open circles), 1 µg/mL (closed triangles), 2 µg/mL (open diamonds), 3 µg/mL (closed squares), 4 µg/mL (open inverted triangles), and 5 µg/mL (closed circles). (C) The data were fitted to a simple binding isotherm to obtain apparent binding constants (K_a_) for PAT-SM6 binding as a function of the coating concentration of LDL (open circles) or oxidized LDL (closed circles). Error bars represent the 95% confidence intervals from the fitting of the ELISA data to a Langmuir binding curve.

ELISA experiments were performed using a range of PAT-SM6 concentrations and plates coated with different concentrations of either LDL or oxidized LDL (Figure 3). The results show a systematic increase in the ELISA signal as a function of PAT-SM6 concentration with more extensive binding observed at higher antigen coating concentrations. The data was fitted to a simple Langmuir binding isotherm to obtain the apparent binding constants (K_a_) as a function of LDL coating concentration (Figure 3C). The results show that the interaction of PAT-SM6 with LDL or oxidized LDL is similar and relatively independent of coating concentrations with apparent K_a_ values approximately 50 µM^−1^, corresponding to dissociation constants of 20 nM. These values indicate that the interaction between PAT-SM6 and LDL is weaker than the interaction between PAT-SM6 and GRP78, where ELISA studies yielded apparent dissociation constants of approximately 4 nM [4].

### The Simultaneous Binding of LDL and GRP78 to PAT-SM6

**Figure 4:**
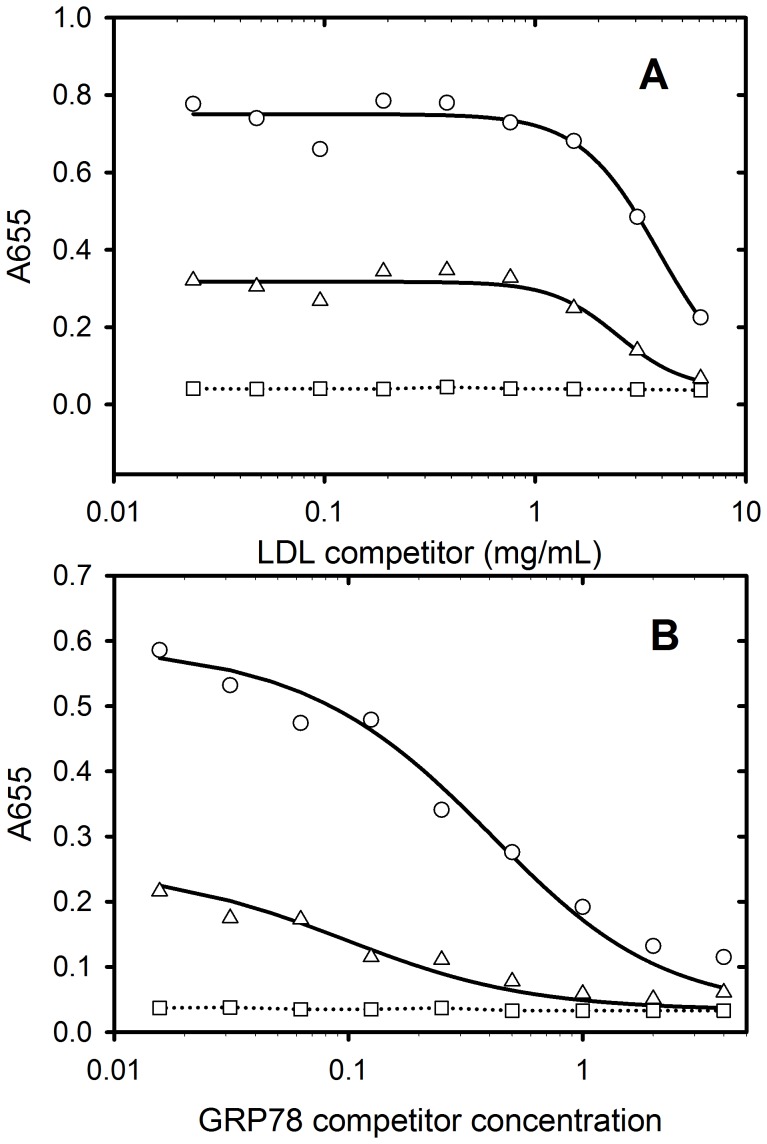
Competition ELISA data for PAT-SM6 obtained using (A) soluble LDL or (B) GRP78 as competitors. Wells were coated with 7.5 mg/mL GRP78 (circles), 7.5 mg/mL LDL (triangles) or were left uncoated (squares). PAT-SM6 (5 mg/mL) was incubated with various concentrations of competitor prior to application to the microtiter plate.

The ability of PAT-SM6 to interact simultaneously with GRP78 and LDL was examined using competition ELISA experiments. The results were performed using low plating concentrations of GRP78 or LDL to allow more efficient inhibition of PAT-SM6 binding by the addition of soluble antigen. Results using soluble LDL as a competitor indicated that significant inhibition of PAT-SM6 binding was observed for plates coated with either GRP78 or LDL. Conversely, results using GRP78 as a competitor revealed significant inhibition of PAT-SM6 binding to immobilised GRP78 or LDL, confirming the ability of GRP78 and LDL to compete with each other for the binding to PAT-SM6. A further observation from Figure 4 is that higher concentrations of LDL are required for half maximal inhibition, compared with GRP78, consistent with the higher affinity of GRP78 for PAT-SM6 revealed from direct ELISA experiments (Figure 3 and [4]).

### Sedimentation Velocity Analysis of PAT-SM6 and LDL

**Figure 5:**
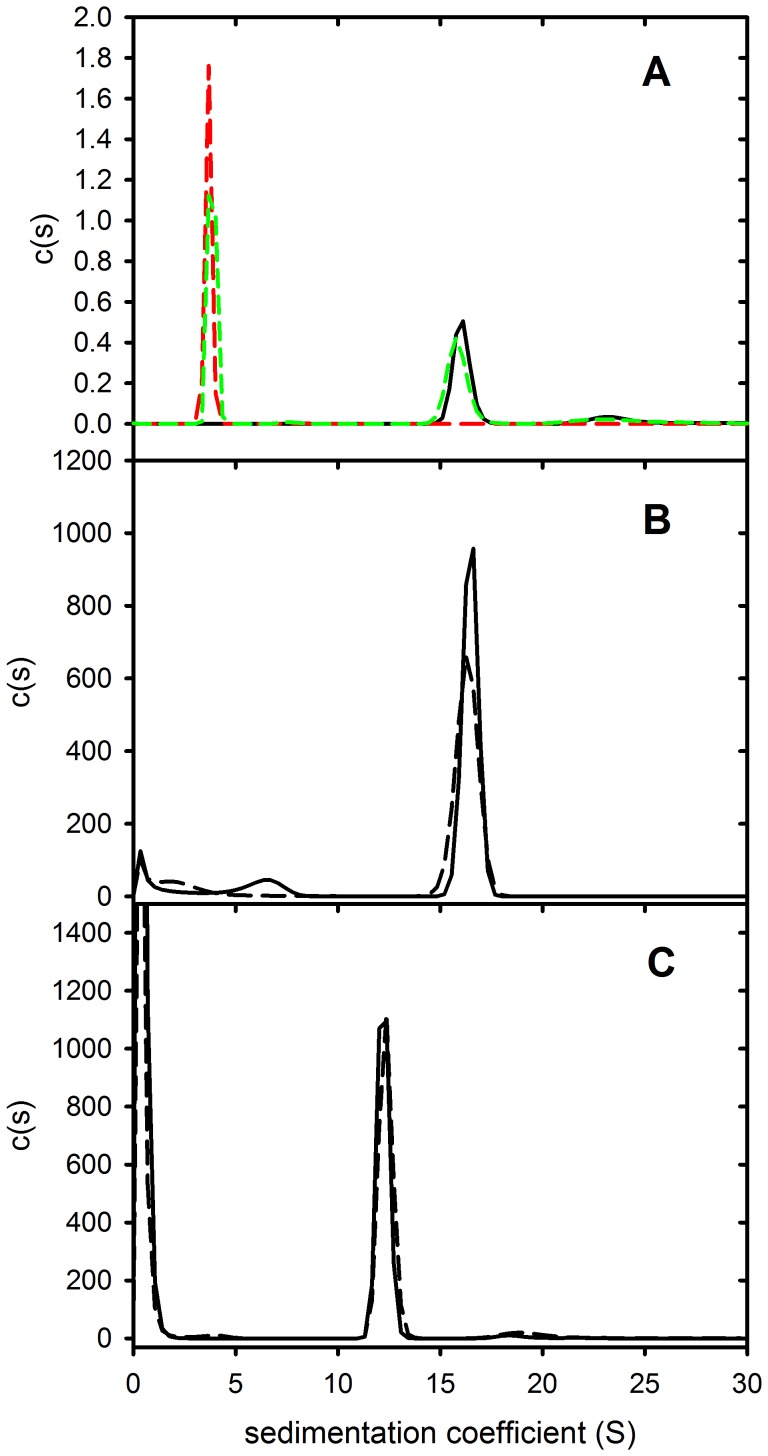
Sedimentation velocity analysis of the PAT-SM6 interaction with LDL. (A) Sedimentation coefficient distributions obtained using absorbtion optics at 280 nm for PAT-SM6 (0.3 µM, black), LDL (1.8 µM, red) and a mixture of PAT-SM6 and LDL (0.3 µM and 1.8 µM, respectively (green). (B) Sedimentation coefficient distributions obtained using fluorescence optics for FITC-labelled PAT-SM6 (40 nM) in the presence (solid line) and absence (broken line) of LDL (1.8 µM). (C) Sedimentation coefficient distributions obtained in the presence of BSA (50 mg/mL) using fluorescence optics for FITC-labelled PAT-SM6 (40 nM) in the presence (solid line) and absence (broken line) of LDL (1.8 µM).

The results in Figure 3 showed that the apparent K_a_ values for the interaction of PAT-SM6 with immobilized LDL or oxidized LDL was independent of the antigen coating concentration. These findings are in contrast with the results obtained with GRP78 [4] where the strong dependence of PAT-SM6 binding on the coating concentration of GRP78 was taken as evidence for antigen clustering as a major determinant of the strength of PAT-SM6 binding interactions. The results observed for LDL suggested that LDL may be clustered on the microtiter plate, even at low plating concentrations, or that PAT-SM6 may bind directly to individual LDL particles. To examine the latter possibility, sedimentation velocity experiments were carried out to characterize the interaction between soluble LDL and PAT-SM6. The results in Figure 5A show sedimentation coefficient distributions for a mixture of LDL and PAT-SM6. Two major peaks are observed with modal sedimentation coefficients close to the values observed for LDL alone and PAT-SM6 alone. Analysis of the average sedimentation coefficient of the faster peak, correcting for the small effect of LDL on solution density and viscosity, revealed no significant difference compared to the average sedimentation coefficient for PAT-SM6 alone. The absence of any detectable change in the rate of sedimentation of PAT-SM6 in the presence of LDL indicates very little interaction between these species under these conditions and is at variance with the strong interactions observed in ELISA experiments (Figures 3 and 4). One possibility considered was that the use of 50 mg/mL BSA as a blocking agent in the ELISA experiments may act as a macromolecular crowding agent and increase the binding affinity [18]. To test this possibility sedimentation velocity experiments were performed using fluorescently labelled PAT-SM6 and the fluorescence detection system in the analytical ultracentrifuge. This allowed the rate of sedimentation of labelled PAT-SM6 to be monitored in the presence and absence of high concentrations of BSA. The results in Figure 5 show that LDL has very little effect on the rate of sedimentation of fluorescently labelled PAT-SM6 either in the presence or absence of 50 mg/mL BSA. The sedimentation velocity results in Figure 5 exclude a high affinity interaction between soluble LDL and individual sites on PAT-SM6 and support the proposal that the binding of PAT-SM6 to LDL immobilised on microtiter plates is driven by an avidity-based binding mechanism of otherwise weak interactions (at least high µM range).

### The Interactions of PAT-SM6 with Antigen-coated Polystyrene Microspheres

**Figure 6:**
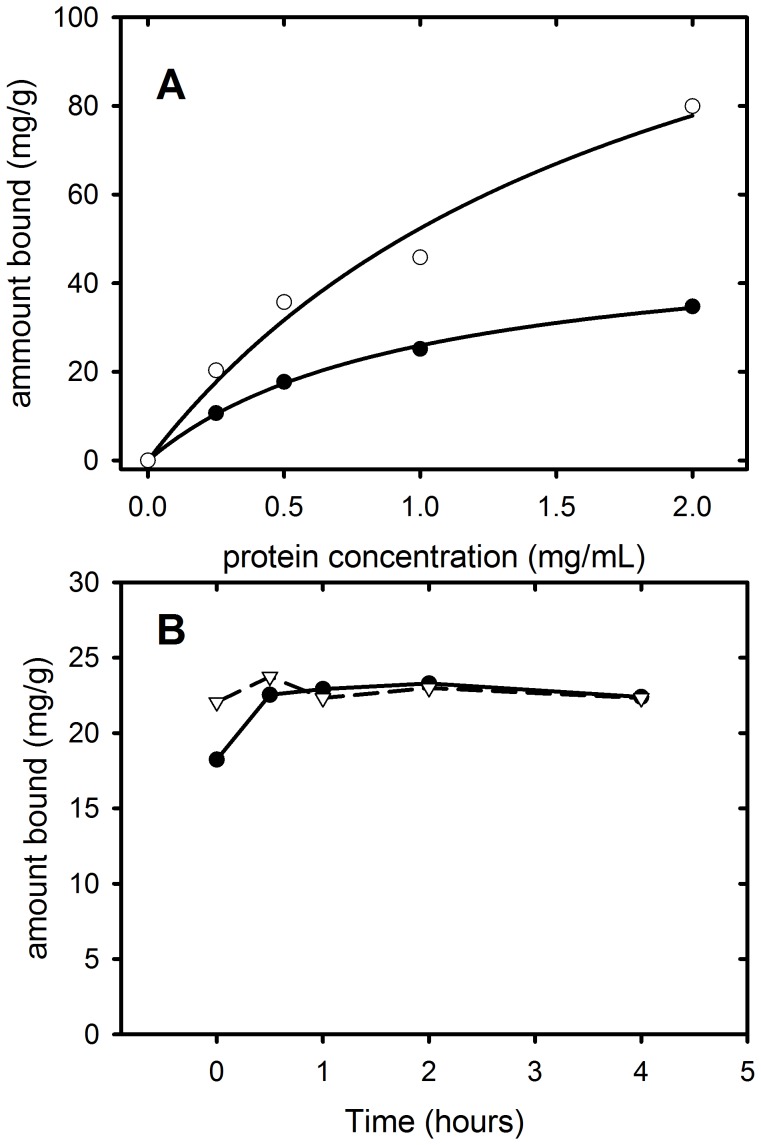
Binding of GRP78 to polystyrene microspheres. (A) Amount of protein bound per gram of microspheres as a function of GRP78 (closed circles) or BSA concentration (open circles). (B) Timecourse of GRP78 (solid circles) and LDL (open triangles) binding to polystyrene microspheres.

The evidence the PAT-SM6 interacts by multi-point attachment to clustered antigens suggested that the absorption of GRP78 or LDL to polystyrene microspheres may increase the ability of these antigens to inhibit PAT-SM6 binding interactions. Antigen-coated polystyrene microbeads were prepared by incubation of polystyrene microbeads with antigens and monitoring the extent of binding using a centrifuge pelleting assay. The results in Figure 6 show that GRP78 and LDL bind rapidly to the polystyrene beads. The results also show the extent of binding of GRP78 increases as a function of GRP78 concentration and that BSA binds to the beads in a saturable manner (Figure 6).

**Figure 7:**
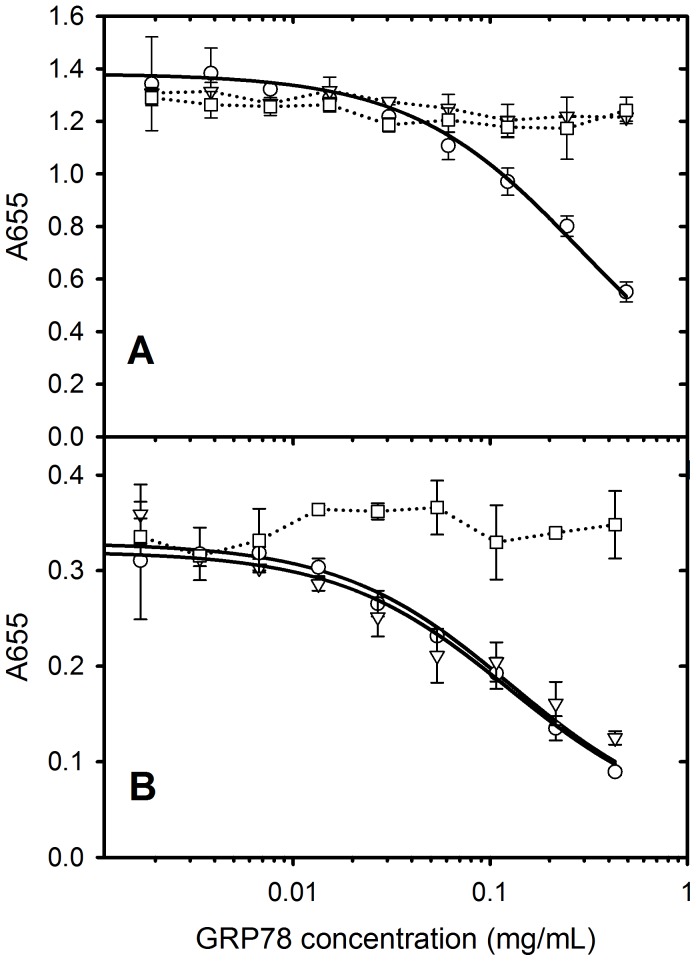
Competition ELISA data for the binding of PAT-SM6 to microtiter plates coated with (A) GRP78 (30 µg/mL) or (B) LDL (30 µg/mL). PAT-SM6 (5 mg/mL) was incubated with GRP78-coated microspheres (circles), soluble GRP78 (triangles) or BSA coated microspheres (squares) prior to addition to the microtiter plates.

**Figure 8:**
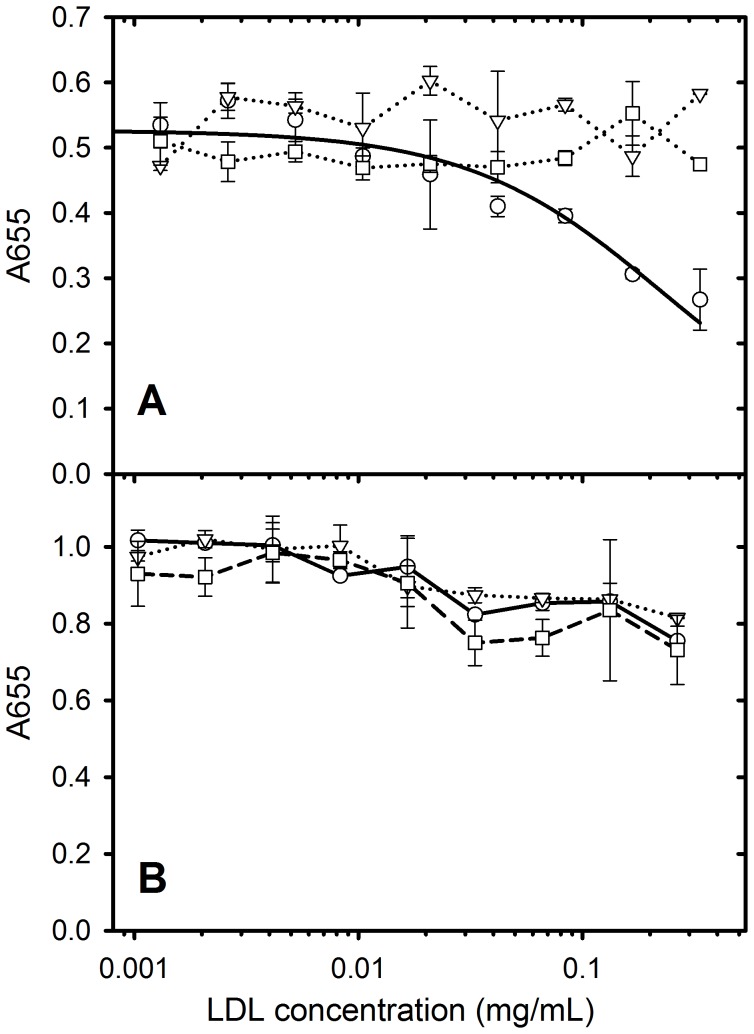
Competition ELISA data for the binding of PAT-SM6 to microtiter plates coated with (A) LDL (30 µg/mL) or (B) GRP78 (30 µg/mL). PAT-SM6 (5 mg/mL) was incubated with LDL-coated microspheres (circles), soluble LDL (triangles) or BSA coated microspheres (squares) prior to addition to the microtiter plates.

ELISA experiments to test whether GRP78 or LDL coated microbeads inhibit the binding of PAT-SM6 to antigen-coated microtiter plates were performed using high concentrations of coating antigen to maximise the extent of clustering on the plate. The results in Figure 7 show that GRP78 coated microspheres inhibit PAT-SM6 binding to microtiter plates coated with either GRP78 while, over the same concentration range and conditions, soluble GRP78 or BSA-coated control microspheres did not inhibit. These results indicate strong binding of PAT-SM6 to GRP78-coated beads via multivalent attachment that sequesters PAT-SM6 and prevents the binding of PAT-SM6 to GRP78 coated microtiter plates. Similar results were obtained using LDL-coated microtiter plates, although in this case both GRP78-coated beads and soluble GRP78 inhibited PAT-SM6 binding to the plates. This result is consistent with the higher apparent avidity of PAT-SM6 for GRP78 compared to LDL. The results in Figure 8 show that LDL coated microspheres inhibit PAT-SM6 binding to microtiter plates coated with LDL, while under the same concentration range and conditions soluble LDL or BSA-coated control microspheres did not inhibit. The results in Figure 8 also show the LDL-coated microspheres did not inhibit the binding of PAT-SM6 to GRP78 coated plates, consistent with a higher avidity of PAT-SM6 for clustered GRP78 compared to clustered LDL.

## Discussion

Natural IgM antibodies form part of the innate immune response where they are involved in the recognition of foreign particles including oxidized and chemically modified proteins and transformed cells [19]. This class of antibody typically show low antigen specificity with the ability to bind more than one type of antigen [20]. This ability to bind more than one type of antigen is evident in the case of PAT-SM6 where ELISA data indicates strong interactions with the structurally unrelated cell membrane-associated GRP78 in tumour cells [3] and with both native and oxidized plasma low density lipoproteins [2]. Previous studies show that the interaction of PAT-SM6 with GRP78 is mediated through an avidity-based mechanism based on the multivalency of the antibody. The results presented in the present study suggest a similar avidity-based mechanism operates for the binding of PAT-SM6 with LDL. Several observations support this conclusion. The interaction of PAT-SM6 with LDL analysed by sedimentation velocity revealed no significant change in the rate of sedimentation of PAT-SM6 in the presence of LDL. In contrast, strong interactions between immobilized GRP78 and PAT-SM6 were observed in ELISA experiments. These studies suggest relatively weak interactions (in the micromolar concentration range) when PAT-SM6 and LDL are free in solution, but much stronger interactions with LDL clustered on the microtiter plate. Support for the role of clustering is also provided by the results from experiments employing antigen-coated polystyrene microspheres, which showed enhanced inhibition of the binding of PAT-SM6 to antigen coated microtiter plates, compared to equivalent concentrations of soluble forms of the antigen. A marked difference with PAT-SM6 interactions with LDL compared to the interactions with GRP78 is that the apparent avidity for PAT-SM6 interactions with LDL did not reveal a dependence on the coating concentration of LDL. In the case of GRP78 the apparent avidity for PAT-SM6 increases with coating concentration, consistent with an antigen clustering effect [4]. A possible explanation for this difference is the large size of LDL and stronger binding to the microtiter plate leading to clustering, even at low coating concentrations.

Analysis of the binding of PAT-SM6 to LDL and oxidized LDL indicated similar apparent avidities (K_d_ approximately 20 nM) and overall weaker binding compared with the interaction with GRP78. Previous studies of the interaction of PAT-SM6 with LDL and oxidized LDL, performed using a single antigen coating and PAT-SM6 concentration yielded a lower ELISA signal (65%) for the binding to LDL compared with oxidized LDL [2] in contrast to the results presented in Figure 3, where the binding curves for PAT-SM6 to LDL and oxidized LDL were very similar. Possible explanations for this discrepancy are variations in the methods used for preparing oxidized LDL. The more significant observation from both studies is the ability of PAT-SM6 to recognise both native LDL and oxidized LDL. The current hypothesis for the killing action of PAT-SM6 on tumour cells is that cell death occurs via an apoptotic pathway accompanied by intracellular lipid accumulation [2]. The present study demonstrates the ability of LDL and GRP78 to compete for binding sites on PAT-SM6 data, providing strong evidence that these proteins share binding sites on the antibody. The multivalent nature of PAT-SM6 would therefore permit this antibody to bind LDL and GRP78 simultaneously, consistent with a mechanism where PAT-SM6 binds plasma LDL and targets tumour cells by binding to GRP78 which is over-expressed externally on the cell surface of tumour cells [3].

## References

[1] Pohle T, Brandlein S, Ruoff N, Muller-Hermelink HK, Vollmers HP (2004) Lipoptosis: tumor-specific cell death by antibody-induced intracellular lipid accumulation. Cancer Res 64: 3900–3906. 15173000 10.1158/0008-5472.CAN-03-3149

[2] Brandlein S, Rauschert N, Rasche L, Dreykluft A, Hensel F, et al (2007) The human IgM antibody SAM-6 induces tumor-specific apoptosis with oxidized low-density lipoprotein. Mol Cancer Ther 6: 326–333. 17237291 10.1158/1535-7163.MCT-06-0399

[3] Rauschert N, Brandlein S, Holzinger E, Hensel F, Muller-Hermelink HK, et al (2008) A new tumor-specific variant of GRP78 as target for antibody-based therapy. Lab Invest 88: 375–386. 18268478 10.1038/labinvest.2008.2

[4] Rosenes Z, Mulhern TD, Hatters DM, Ilag LL, Power BE, et al (2012) The Anti-Cancer IgM Monoclonal Antibody PAT-SM6 Binds with High Avidity to the Unfolded Protein Response Regulator GRP78. PLoS One 7: e44927. 23028685 10.1371/journal.pone.0044927 PMC3446985

[5] Tchoudakova A, Hensel F, Murillo A, Eng B, Foley M, et al (2009) High level expression of functional human IgMs in human PER.C6® cells. mAbs 1: 163–171. 20061826 10.4161/mabs.1.2.7945 PMC2725415

[6] Gagnon P, Hensel F, Richieri R (2008) Purification of IgM Monoclonal Antibodies: Manufacturing challenges surround the use of IgM monoclonal. BioPharm Int Sup: 26–36.

[7] Havel RJ, Eder HA, Bragdon JH (1955) The distribution and chemical composition of ultracentrifugally separated lipoproteins in human serum. The Journal of clinical investigation 34: 1345–1353. 13252080 10.1172/JCI103182 PMC438705

[8] Stewart CR, Tseng AA, Mok YF, Staples MK, Schiesser CH, et al (2005) Oxidation of low-density lipoproteins induces amyloid-like structures that are recognized by macrophages. Biochemistry 44: 9108–9116. 15966734 10.1021/bi050497v

[9] Winder AF, Gent WL (1971) Correction of light-scattering errors in spectrophotometric protein determinations. Biopolymers 10: 1243–1251. 5095095 10.1002/bip.360100713

[10] Fless GM, Scanu AM (1975) Physicochemical characterization of Rhesus low density lipoproteins. Biochemistry 14: 1783–1790. 164895 10.1021/bi00679a034

[11] Petoukhov MV, Franke D, Shkumatov AV, Tria G, Kikhney AG, et al (2012) New developments in the ATSAS program package for small-angle scattering data analysis. Journal of Applied Crystallography 45: 342–350. 25484842 10.1107/S0021889812007662 PMC4233345

[12] Schuck P (2000) Size-distribution analysis of macromolecules by sedimentation velocity ultracentrifugation and lamm equation modeling. Biophysical journal 78: 1606–1619. 10692345 10.1016/S0006-3495(00)76713-0 PMC1300758

[13] Esterbauer H, Jurgens G, Quehenberger O, Koller E (1987) Autoxidation of human low density lipoprotein: loss of polyunsaturated fatty acids and vitamin E and generation of aldehydes. Journal of lipid research 28: 495–509. 3598395

[14] Atkinson D, Deckelbaum RJ, Small DM, Shipley GG (1977) Structure of human plasma low-density lipoproteins: molecular organization of the central core. Proceedings of the National Academy of Sciences of the United States of America 74: 1042–1046. 191827 10.1073/pnas.74.3.1042 PMC430581

[15] Baumstark MW, Kreutz W, Berg A, Frey I, Keul J (1990) Structure of human low-density lipoprotein subfractions, determined by X-ray small-angle scattering. Biochimica et biophysica acta 1037: 48–57. 2294970 10.1016/0167-4838(90)90100-t

[16] Meyer DF, Nealis AS, Macphee CH, Groot PH, Suckling KE, et al (1996) Time-course studies by synchrotron X-ray solution scattering of the structure of human low-density lipoprotein during Cu(2+)-induced oxidation in relation to changes in lipid composition. The Biochemical journal 319 (Pt 1): 217–227. 10.1042/bj3190217 PMC1217758 8870672

[17] Meyer DF, Mayans MO, Groot PH, Suckling KE, Bruckdorfer KR, et al (1995) Time-course studies by neutron solution scattering and biochemical assays of the aggregation of human low-density lipoprotein during Cu(2+)-induced oxidation. The Biochemical journal 310 (Pt 2): 417–426. 10.1042/bj3100417 PMC1135911 7654177

[18] Minton AP (2000) Implications of macromolecular crowding for protein assembly. Current Opinion in Structural Biology 10: 34–39. 10679465 10.1016/s0959-440x(99)00045-7

[19] Vollmers HP, Brandlein S (2009) Natural antibodies and cancer. N Biotechnol 25: 294–298. 19442595 10.1016/j.nbt.2009.03.016

[20] Avrameas S (1991) Natural autoantibodies: from ‘horror autotoxicus’ to ‘gnothi seauton’. Immunology today 12: 154–159. 1715166 10.1016/S0167-5699(05)80045-3

